# A Novel Implementation of the LDEM in the Ansys LS-DYNA Finite Element Code

**DOI:** 10.3390/ma14247792

**Published:** 2021-12-16

**Authors:** Andrea Zanichelli, Angélica Colpo, Leandro Friedrich, Ignacio Iturrioz, Andrea Carpinteri, Sabrina Vantadori

**Affiliations:** 1 Department of Engineering & Architecture, University of Parma, Parco Area delle Scienze 181/A, 43124 Parma, Italy; andrea.zanichelli@unipr.it (A.Z.); andrea.carpineri@unipr.it (A.C.); 2 Mechanical Post-Graduate Program, Federal University of Rio Grande do Sul, Sarmento Leite 425, Porto Alegre CEP 90050-170, Brazil; angelicabcolpo@gmail.com (A.C.); 00107169@ufrgs.br (I.I.); 3 Department of Mechanical Engineering, Federal University of Pampa, Tiaraju 810, Alegrete CEP 97546-550, Brazil; leandroffriedrich@gmail.com

**Keywords:** Ansys LS-DYNA, bending, fracture, LDEM, sandwich panels

## Abstract

In this paper, a novel implementation of the Lattice Discrete Element Method (LDEM) is proposed: in particular, the LDEM is implemented in the Ansys LS-DYNA finite element code. Such an implementation is employed to evaluate the fracture behaviour of sandwich panels under bending. First, the novel hybrid model proposed is validated by simulating some three-point bending experimental tests carried out at the University of Parma, and then it is used to model the fracture behaviour of sandwich panels under four-point bending. Failure mechanisms, damage locations, and load-deflection curves are numerically determined by employing such a novel model, and the results show a good agreement with the available experimental findings.

## 1. Introduction

Composite materials consist of at least two phases with different physical, mechanical, and chemical properties, which are combined in order to improve a specific property [1]. In the last decades, composite materials have become increasingly used in many engineering applications, due to their improved properties with respect to those of traditional materials.

Three different categories of composite materials may be distinguished [2]: fibre-reinforced materials, particle-reinforced materials, and layered materials.

Relatively to both fibre-reinforced and particle-reinforced materials, one or more types of fibres or particles (reinforcing phase) are employed in order to improve either mechanical or physical properties of a specific base material (matrix). A reinforcing phase made of steel, glass, carbon, synthetic, or natural fibres is commonly embedded in a matrix that could be, for example, either cementitious or polymeric [3,4,5,6,7,8,9].

As far as layered materials are concerned, several layers made of different materials are stacked, and may be characterised by different orientations and thickness [10,11,12,13]. Each layer or group of layers is devoted to a specific task, ranging from structural strength to insulation or lightening.

Among layered materials, sandwich panels are noteworthy as they are generally light, with excellent properties in terms of load-bearing capacity, thermal and acoustic insulation, and waterproofing. They consist of a soft, thick core between two thin sheets held together by means of adhesive layers. The core, which may be characterised by either a continuous or a discrete periodic geometry (such as honeycomb and corrugated cores), plays an important role in sandwich panels [12]. For instance, a higher rigidity of the entire composite can be achieved by increasing the thickness of the core, with a consequent slight increase in terms of weight [14]. Moreover, the use of specific materials as core materials (such as rock wool) may provide good fireproof performance.

Nowadays, sandwich panels are widespread in the fields of both aerospace [15], shipbuilding [16], automotive [14], civil engineering [12], and furniture industry [17]. Extensive experimental research has been recently carried out in order to understand the mechanical behaviour of such materials subjected to specific loadings, such as through-thickness compression [18], longitudinal compression [19], bending moment [20], torsion [21], indentation [22], and dynamic loading [23].

Due to the large number of variables affecting the properties of sandwich panels, numerical models are generally employed for design practises. For instance, finite element models have been recently used by Bunyawanichakul et al. [24] to simulate the failure modes of a local-reinforced sandwich structure. Moreover, Styles et al. [25] analysed the influence of core thickness on the flexural behaviour of a composite sandwich structure with an aluminium foam core.

The Finite Element Method (FEM) has the advantage of a great versatility in modelling both complex geometries and nonlinearities involved in the process. The FE models are particularly useful for structures which do not contain discontinuities (for example, cracks). In order to simulate bodies characterised by discontinuities, some strategies exist for employing FEM, for example, the cohesive interfaces method [26] and the extended finite element method [27]. However, FEM is not able to accurately simulate the dynamic process of damage nucleation and propagation, and thus to predict the nonlinear deformation and failure modes of sandwich panels [28].

When the material damaging (in terms of cracks nucleation, propagation, and interactions) needs to be taken into account, numerical models based on the Discrete Element Method (DEM) have proved to be more suitable than FEM. In more detail, the appearance of discontinuities within the materials is considered in a natural way as a degradation of the interactions among elements in the DE model, without any need to introduce external criteria.

In the present research work, the Lattice Discrete Element Method (LDEM) originally proposed by Riera [29] is employed to evaluate the fracture behaviour of sandwich panels subjected to bending. Note that the novelty of the paper is represented by the hybrid model proposed (named LDEM-DYNA model), obtained by using Ansys LS-DYNA finite element code [30] in conjunction with the LDEM [31,32,33,34,35,36,37,38,39,40,41].

In more detail, the LDEM model employed consists of a set of masses joined by bar elements, characterised by a regular cubic arrangement. Moreover, the bars have a bilinear constitutive law, based on the Hillerborg model [42], which allows the breaking of the bar elements themselves, thus taking into account the degradation of the material.

The paper is organised as follows. First, the basic concepts of the LDEM together with the details of implementation in the Ansys LS-DYNA finite element code are described in Section 2 and Section 3, respectively. Subsequently, Section 4 is devoted to discussing the experimental three-point bending tests carried out at the University of Parma on sandwich panels. Then, the LDEM-DYNA model is employed to simulate the above experimental campaign, as well as some four-point bending tests (Section 5). Finally, conclusions are summarised in Section 6, highlighting that the novel hybrid model proposed allows us to obtain quite satisfactory results in terms of failure mechanisms, damage locations, and load-deflection curves.

## 2. Lattice Discrete Element Method Description

**Figure 1:**
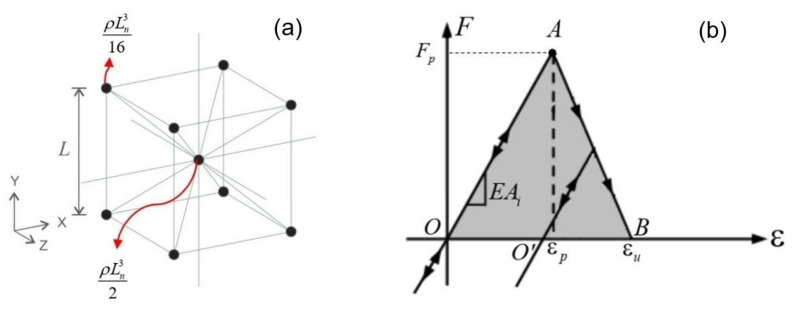
LDEM: (**a**) Basic cubic module employed in the discretisation and (**b**) bilinear constitutive law attributed to the bars.

In Lattice Discrete Element Method (LDEM), the continuum medium is represented by a cubic arrangement of trusses in which the total mass is concentrated at the nodes [29]. Each node has three degrees of freedom, which correspond to the nodal displacements in the three directions of an orthogonal coordinate system (XYZ in Figure 1a). The discretisation strategy employs a basic cubic module with twenty bars (commonly named elements) and nine nodes, as is shown in Figure 1a. The lengths of the longitudinal and diagonal elements are $L_{n}=L$ and $L_{d}=\left(\sqrt{3}/2\right)L$, respectively [43], where L is the length of the cubic module (Figure 1a).

The total mass of the basic cubic module is equal to $m=\rho L_{n}{}^{3}$, with ρ being the mass density of the material and $L_{n}{}^{3}$ the volume of the module. It is assumed that the total mass is discretised in the nodes (Figure 1a): half of the mass (equal to $\rho L_{n}{}^{3}/2$) is concentrated at the central node, whereas the other half is equally distributed among the eight nodes of the vertices ($\rho L_{n}{}^{3}/16$ at each of such eight nodes).

In the case of an isotropic elastic material, the stiffness of the bars is assumed to be the same as the continuum. Therefore, the cross-section area $A_{n}$ of each longitudinal element is given by:

(1) $$A_{n}=\frac{1}{2(1+\nu )}L_{n}{}^{2}$$

where ν is the Poisson ratio of the material, whereas the cross-section area $A_{d}$ of each diagonal element is given by:

(2) $$A_{d}=\frac{2}{\sqrt{3}}\delta A_{n}$$

with δ equal to $\delta =9\nu /\left(4-8\nu \right)$.

Note that the equivalence with the isotropic continuum is complete for ν=0.25; on the other hand, a difference arises in terms of shear for ν≠0.25, as is detailed in [44]. Such a difference is very small, and consequently can be neglected in the case of 0.20≤ν≤0.30. For ν values outside such a range, a different array of elements must be used to form the basic modulus [45]. Details on the calculation of the equivalent cross-section area for both longitudinal and diagonal bars can be found in [45].

The system of equations resulting from applying the Newton’s second law to each node is given by:

(3) $$M_{ij}{\ddot{x}}_{j}+C_{ij}{\dot{x}}_{j}+F_{i}\left(t\right)-P_{i}\left(t\right)=0$$

where the vectors ${\ddot{x}}_{j}$ and ${\dot{x}}_{j}$ represent the nodal acceleration and velocity, respectively; $M_{ij}$ and $C_{ij}$ are the mass and damping matrices, respectively; and the vectors $F_{i}\left(t\right)$ and $P_{i}\left(t\right)$ are the internal and external nodal forces, respectively. As matrices $M_{ij}$ and $C_{ij}$ are diagonal, the Equation (3) are not coupled, and can be easily integrated in the time domain using an explicit finite difference scheme. In this way, the nodal coordinates are updated at each time step, and thus large displacements are accounted for without introducing any modification in the formulation [46]. By considering such an aspect, discrete element method is different from finite element method, in which a suitable nonlinear formulation, such as Lagrangian or total Lagrangian [47], should be employed.

Furthermore, the maximum time-interval for integration, $\Delta t_{max}$, can be determined by applying the Courant–Friedrich–Lewy criterion [48]:

(4) $$\Delta t_{max}\leq \frac{L_{d}}{C_{p}}$$

where $C_{p}$ is defined as follows:

(5) $$C_{p}=\sqrt{E/\rho }$$

with E being the Young’s modulus of the material. The relationship (4) between the maximum time-interval and the element length is important to be satisfied for the numerical stability of the integration scheme.

The convergence of the solution using LDEM for both linear elastic problems and elastic stability problems was verified by different researchers as is reported in [49], where a strategy to provide a consistent equivalence between different regular lattice arrangements and the represented solid was proposed.

When a bar breaks, that is, the bar strain reaches its ultimate value $\varepsilon_{u}$, an equivalent fracture area is generated, causing a release of fracture energy. Such an energy depends on both the fracture area and the material constitutive law. Different shapes of the constitutive law may be assumed for the bars. More precisely, the law proposed for quasi-brittle materials [42] was employed in [29,50], so that the LDEM could be applied to solve brittle fracture problems. Another law that can be used is that shown in Figure 1b, which allows to take into account the irreversible effects of crack nucleation and propagation, which produce material plasticity.

The bilinear law shown in Figure 1b directly depends on three local parameters: $EA_{i}$, $\varepsilon_{u}$**,** and $\varepsilon_{p}$. The bar specific stiffness $EA_{i}$ is a function of both the Young’s modulus E and the cross-section area of the bar $A_{i}$, where the subscript i is equal to n for normal bar and equal to d for diagonal bar. The ultimate strain $\varepsilon_{u}$ is the strain value for which the element loses its load bearing capacity (that is, the bar breaks), whereas the critical strain $\varepsilon_{p}$ is the strain at the crack initiation.

The value of $\varepsilon_{u}$ is computed by considering the dissipated energy released when the element fails [51]:

(6) $$\int_{0}^{\varepsilon_{u}}F\left(\varepsilon \right)d\varepsilon =\frac{G_{f}\;A_{i}^{*}}{L_{i}}$$

where $F\left(\varepsilon \right)$ is the force in the bar (see Figure 1b), $A_{i}^{*}$ is the equivalent fracture area of the *i*-th element, $G_{f}$ is the fracture energy, and $L_{i}$ is the element length. As the dissipated energy is represented by the area of the OAB triangle in Figure 1b, which is equal to $\varepsilon_{u}\varepsilon_{p}EA_{i}/2$, the ultimate strain for the *i*-th element is given by:

(7) $$\varepsilon_{u}=\frac{G_{f}}{\varepsilon_{p}E}\left(\frac{A_{i}^{*}}{A_{i}}\right)\left(\frac{2}{L_{i}}\right)$$

By equating the fracture energy dissipated by the continuum with its discrete counterpart, the equivalent fracture area $A_{i}^{*}$ of the *i*-th element can be determined: $A_{i}^{*}=\left(\frac{3}{22}\right)L_{i}^{2}$. Details may be found in [46,51].

The value of $\varepsilon_{p}$ is computed as follows. As is well-known, according to the classical fracture mechanics, the material fracture toughness $K_{c}$ is given by [52]:

(8) $$K_{c}=\sigma_{{}_{p}}^{*}Y\sqrt{\pi q}$$

where $\sigma_{{}_{p}}^{*}$ is the critical stress and Y is a parameter that accounts for the influence of boundary conditions, load, and orientation of the critical crack, having a length equal to q. By assuming that the behaviour is linear up to the crack initiation, then $\sigma_{p}=E\varepsilon_{p}$. Note that such a stress value may vary for the different bars forming the model, that is, $\sigma_{p}$ is a local parameter referring to the *i*-th bar.

By recalling the relationship between the fracture toughness $K_{c}$ and the fracture energy $G_{f}$, the following equation is obtained:

(9) $$\sqrt{G_{f}E}=E\varepsilon_{p}Y\sqrt{\pi q}$$

In order to simplify Equation (9), an equivalent length $d_{eq}$ is defined as follows:

(10) $$d_{eq}=q\pi Y^{2}$$

and, by substituting Equation (9) in Equation (10), we get:

(11) $$d_{eq}=\frac{G_{f}}{{\left(\varepsilon_{p}\right)}^{2}E}$$

Equation (11) indicates that $d_{eq}$ may be regarded as a material property, as it does not depend on the discretisation level but represents a characteristic length of the material (similar to the width of the plasticity region at crack tip in the Dugdale model). Therefore, $\varepsilon_{p}$ can be obtained from Equation (11):

(12) $$\varepsilon_{p}=\sqrt{\frac{G_{f}}{d_{eq}E}}$$

Finally, by combining Equation (11) with Equation (7), $\varepsilon_{u}$ may also be expressed as follows:

(13) $$\varepsilon_{u}=\varepsilon_{p}d_{eq}\left(\frac{A_{i}^{*}}{A_{i}}\right)\left(\frac{2}{L_{i}}\right)$$

Under compression, the element behaviour remains linear and elastic (Figure 1b).

As can be seen in Figure 1b, the constitutive law allows to capture the residual deformation due to the initial stiffness elements conservation. Examples of this elementary constitutive law implementation can be found in [44,53].

Some observations can be made on Equation (13). When $\varepsilon_{p}$ is equal to $\varepsilon_{u}$, the minimum area of bilinear constitutive law is obtained, as $\varepsilon_{u}$ has to be larger or equal to $\varepsilon_{p}$ in order to ensure the energy equilibrium into the element. In such a condition, a limit relationship between the equivalent length $d_{eq}$ and the element length $L_{i}$ can be found [51]:

(14) $$d_{eq}\geq 1.439L_{i}$$

The equivalent length $d_{eq}$ can be also determined by exploiting the concept of the stress brittleness number s proposed by Carpinteri [54], given by:

(15) $$s=\frac{K_{c}}{\sigma_{p}R_{e}{}^{1/2}}$$

where $\sigma_{p}$ is the material strength and $R_{e}$ is the structure characteristic size. Note that s is able to represent the structure behaviour, that is, a fragile behaviour is expected for s→0, whereas a ductile behaviour is expected for s→∞.

Equation (15) can be thus rewritten by combining Equations (7), (8), and (13):

(16) $$d_{eq}=s^{2}R_{e}$$

In a LDE model, the material intrinsic inhomogeneity can be implemented by considering a three-dimensional stochastic field for the fracture energy $G_{f}$. In general, the correlation lengths $L_{cx},L_{cy}$, and $L_{cz}$, along the three directions x,y,z, are used to define the linear spatial correlation of the fracture energy. More precisely, a Weibull probability distribution is assumed along each direction. Details may be found in [36].

Note that other properties can also be implemented as random fields, such as the Young’s modulus and the Poisson ratio, as is reported in [55]. Although the parameters $d_{eq}$ and E are constants, $\varepsilon_{p}$ and $\varepsilon_{u}$ are random parameters depending on the $G_{f}$ random field. Therefore, each element is assigned a randomly different bilinear constitutive law. Details about the random field are presented and discussed in [56].

## 3. LDEM Implementation in Ansys LS-DYNA

The LDEM described in Section 2 can be implemented in the Ansys LS-DYNA finite element code [30].

A LDEM-DYNA model allows the use of both discrete elements and finite elements in a unique model, thus creating a hybrid model. More precisely, the region where the fracture is expected to occur is modelled by means of discrete elements, whereas the rest of the body is discretised by using finite elements.

**Figure 2:**
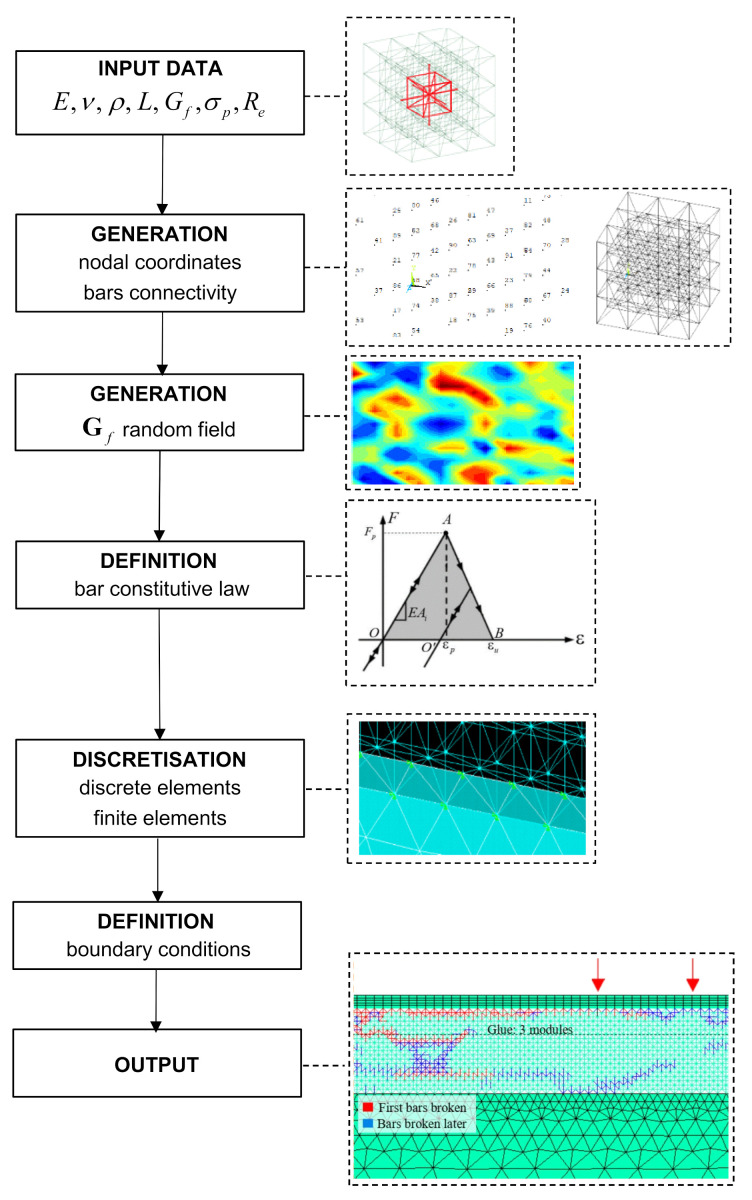
Schematisation of the procedure employed to create the hybrid model.

The main steps to create the hybrid model are summarised in Figure 2 and hereafter detailed.

The regions where Discrete Elements (DEs) are employed is generated firstly. The *input data* needed consist in the bar element length L and some material properties (E, ν, ρ, $G_{f}$, $\sigma_{p}$, $R_{e}$).

Then, the *nodal coordinates and the connectivity of the bars* are generated within the LDEM-DYNA code. The bars that make up the basic modules are modelled in Ansys LS-DYNA by using discrete spring elements, named Explicit Spring-Damper (COMBI165) [30]. Each COMBI165 is a two-node 1-D element, and its behaviour is that of a simple spring or damper system.

Subsequently, a *random field* is introduced for $G_{f}$ and, as a consequence, randomness is indirectly assigned to the critical strain (see Equation (12)). In this way, the maximum stress of each element is also random. Note that $G_{f}$ is the most influential setting on the model response.

The possibility of including the material heterogeneity is also available in a LDEM-DYNA model. The spatial correlation of a random field is defined by means of a correlation length. Details may be found in Refs. [44,56,57].

Then, the *bilinear constitutive law* (see Figure 1b) is attributed to each bar of the model.

To represent a mass/spring system, it is also needed to add a mass element. In a LDEM-DYNA model, the mass of the simulated body is discretised and concentrated in the COMBI165 nodes, where the mass value depends on the node position within the basic module, as is discussed in Section 2. The *mass discretisation* is performed by using the Explicit 3-D Structural Mass (MASS166) element.

After the DEs are built, the FEM region is generated. Note that Explicit 3-D Structural Solid (SOLID164) elements [30] are used for such a region.

Then, LDEM and FEM regions are linked together, thus originating a hybrid model. More precisely, the CPINTF command is used in order to couple the degrees of freedom (displacements and rotations) of coincident nodes, that is, the four nodes of each LDEM cubic module are connected with the four nodes of the corresponding finite element.

Finally, the *boundary conditions* of the problem are defined.

## 4. Experimental Campaign Examined

### 4.1. Testing Apparatus

**Figure 3:**
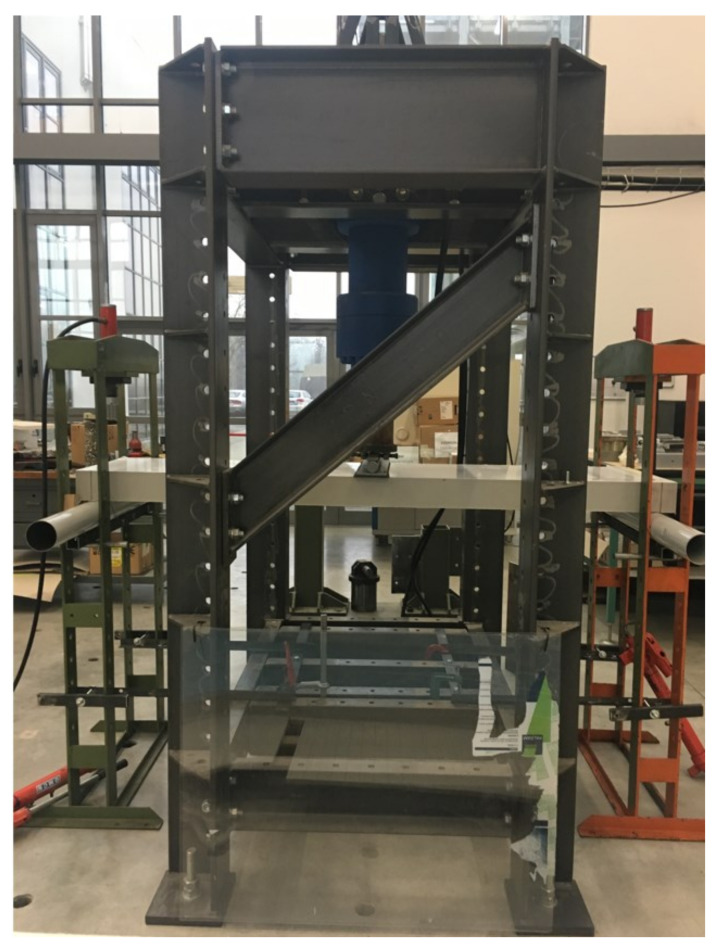
Electronically controlled hydraulic machine employed.

Now, the flexural behaviour of sandwich panels is experimentally examined. Three-point bending tests have been carried out at the Materials and Structures Testing Laboratory of the University of Parma, in accordance with the ASTM C393/C393M, ASTM D7249/D7249M, and ASTM D7250/D7250M standard specifications [58,59,60]. The tests have been performed by means of an electronically controlled hydraulic machine with a capacity of 200 kN (Figure 3).

The tests have been carried out under displacement control. More in detail, a vertical displacement d is imposed by means of a piston with speed equal to 15 mm/min, until the panel failure condition is reached. The vertical load is directly measured by the actuator and recorded automatically during testing.

### 4.2. Specimen Geometry and Material Properties

**Figure 4:**
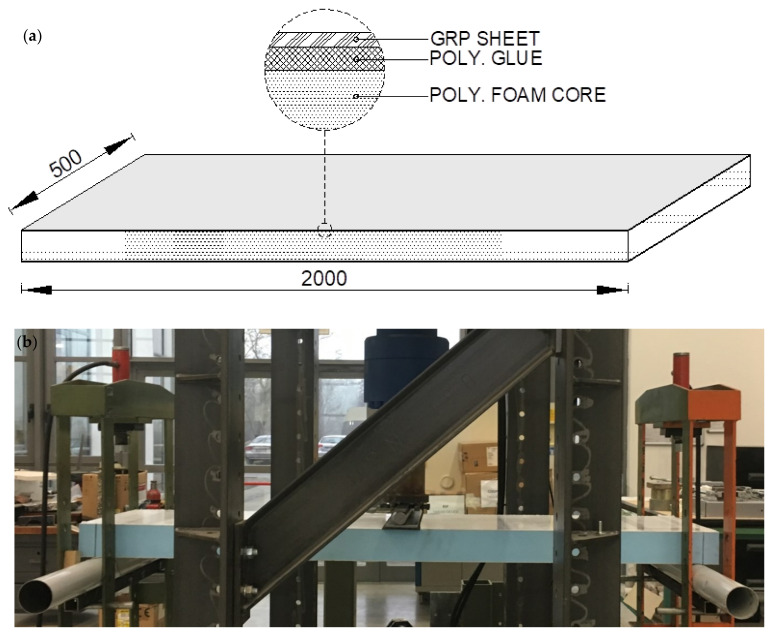
Specimen: (**a**) geometry (sizes in mm) and (**b**) testing setup.

A total number of three specimens (named T1, T2, and T3) are subjected to three-point bending testing. The thickness, width and length of such specimens are equal to 103 mm, 500 mm, and 2000 mm, respectively, and the span between the supports is equal to 1900 mm. In Figure 4, both specimen geometry (Figure 4a) and testing setup (Figure 4b) are shown.

The sandwich panels consist of extruded polystyrene foam core (with a thickness equal to 100 mm) between two thin sheets (also named skins) of fiberglass composite material GRP (each sheet with a thickness equal to 1.5 mm). The Young’s modulus E and the tensile strength $\sigma_{P}$ of the polystyrene foam are equal to 28 MPa and 0.7 MPa, respectively [61]. Moreover, the density ρ of the core is equal to 40 kg/m^3^, whereas the GRP is characterised by a density equal to 1300 kg/m^3^ and an elastic modulus equal to 7 GPa [61]. The skins are coupled to the central core through a two-component polyurethane glue. The glue density is 1500 kg/m^3^, whereas the tensile strength and the Young’s modulus are equal to 13 MPa and 162 MPa, respectively [61].

The material properties of core, sheets, and glue are listed in Table 1.

### 4.3. Experimental Results

**Figure 5:**
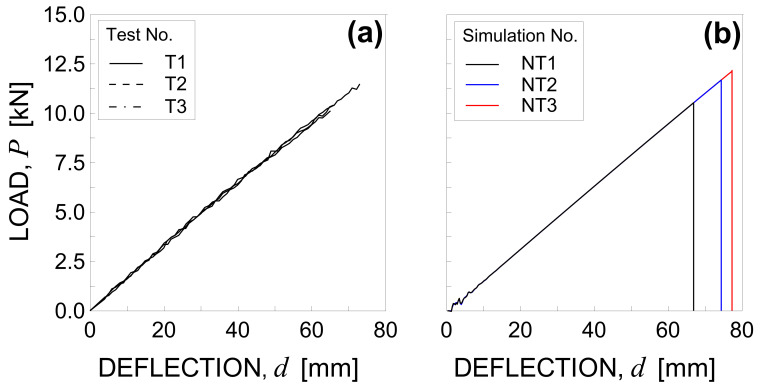
Load-deflection curve under three-point bending: (**a**) experimental results and (**b**) numerical simulations by considering three different $G_{f}$ random fields.

The load-deflection curves obtained are plotted in Figure 5a for each tested specimen.

The maximum load is equal to 11.56 kN, 10.61 kN, and 10.46 kN for test No. T1, T2, and T3, respectively. Such values are reached in correspondence to a vertical displacement d equal to 73.93 mm, 67.92 mm, and 66.94 mm for test No. T1, T2, and T3, respectively. Consequently, the average values of peak load and maximum deflection are 10.88 kN (with a standard deviation equal to 0.49 kN) and 69.60 mm (with a standard deviation equal to 3.09 mm).

Note that sandwich panels subjected to bending loading may be characterised by various failure modes, depending on the geometry of the panel, the properties of the constituent materials and the loading condition [62]. The most common failure modes in sandwich panels are (i) compressive failure of the top skin and tensile failure of the bottom skin, (ii) skin debonding, (iii) skin buckling, (iv) skin indentation of the core, and (v) shear failure of the core. More in detail, skin failure occurs when the ultimate stress of the skin is reached before that of the core. Skin debonding may occur when the adhesive cannot withstand the interfacial shear stress between the skin and the core. Skin buckling may appear at the top compressed side of the panel and is typical of sandwich panels with a foam core. Indentation and core failure occur when the ultimate stress of the core is reached before that of the skin. Note that the final rupture of the panel is generally the result of the interaction of more than one mechanism.

**Figure 6:**
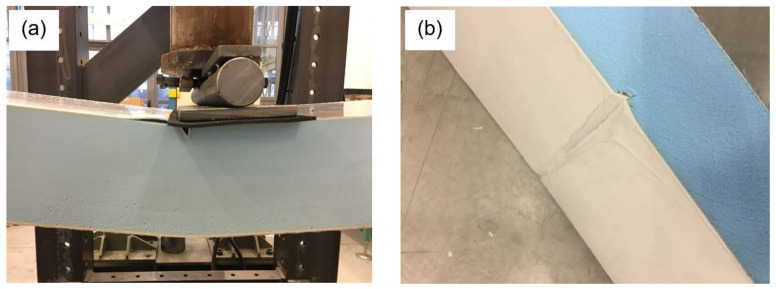

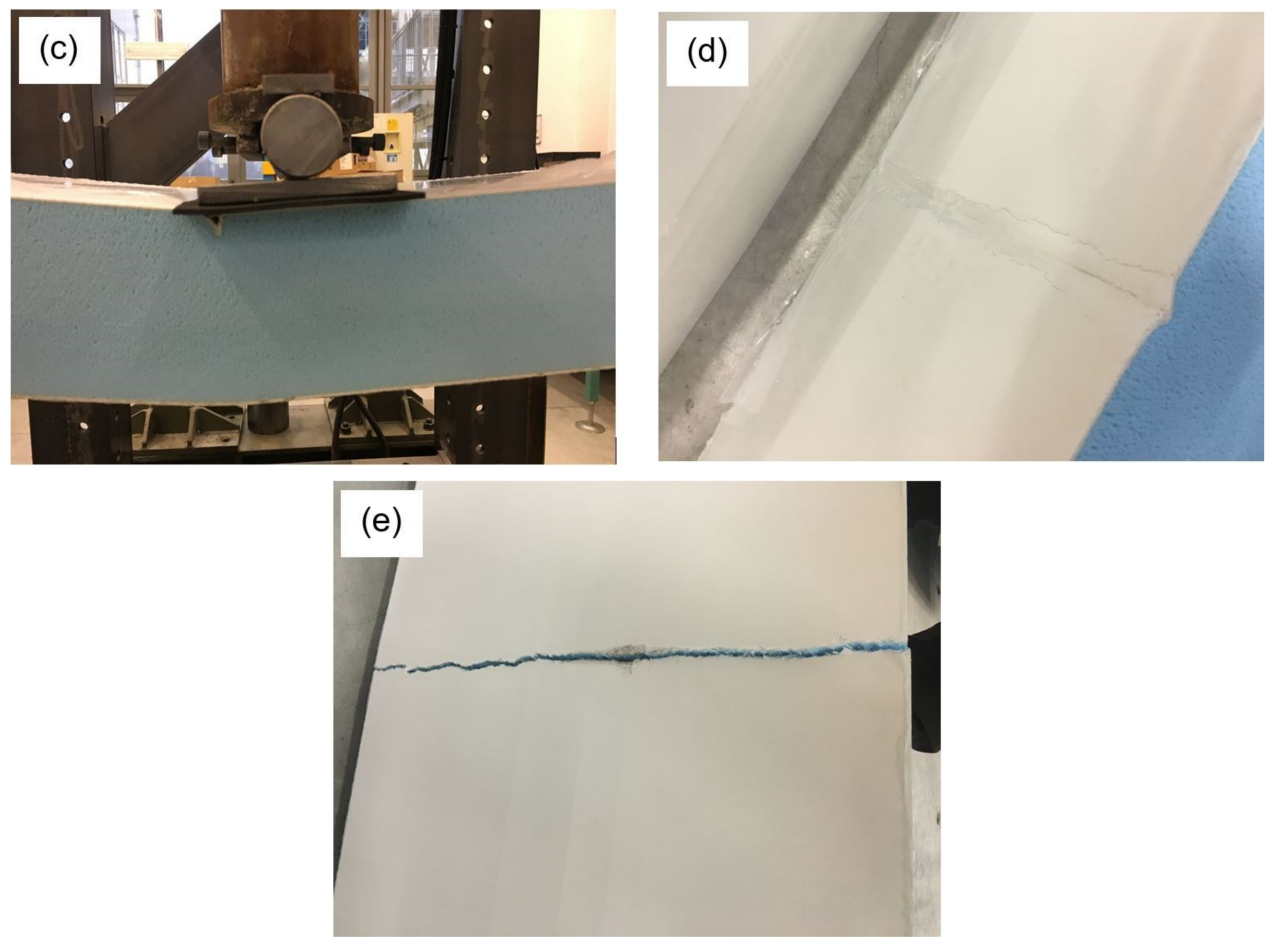
Failure modes observed during the experimental campaign for tests No.: T1 (**a**–**b**), T2 (**c**–**d**), and T3 (**e**).

The failure modes observed during the present experimental campaign are shown in Figure 6. In particular, the final rupture is caused by the core failure for all the specimens. Moreover, the foam core is responsible for other failure mechanisms, that is, indentation (specimens No. T1 and T2, see Figure 6a,c) and buckling (specimen No. T1, see Figure 6a) of the top GRP layer.

## 5. Simulations by LDEM-DYNA

### 5.1. Three-Point Bending Testing

The flexural behaviour of sandwich panels is analysed by means of the LDEM-DYNA numerical model described in Section 3. In particular, the experimental three-point bending tests discussed in Section 4 are hereafter simulated.

#### 5.1.1. Numerical Model Description

**Figure 7:**
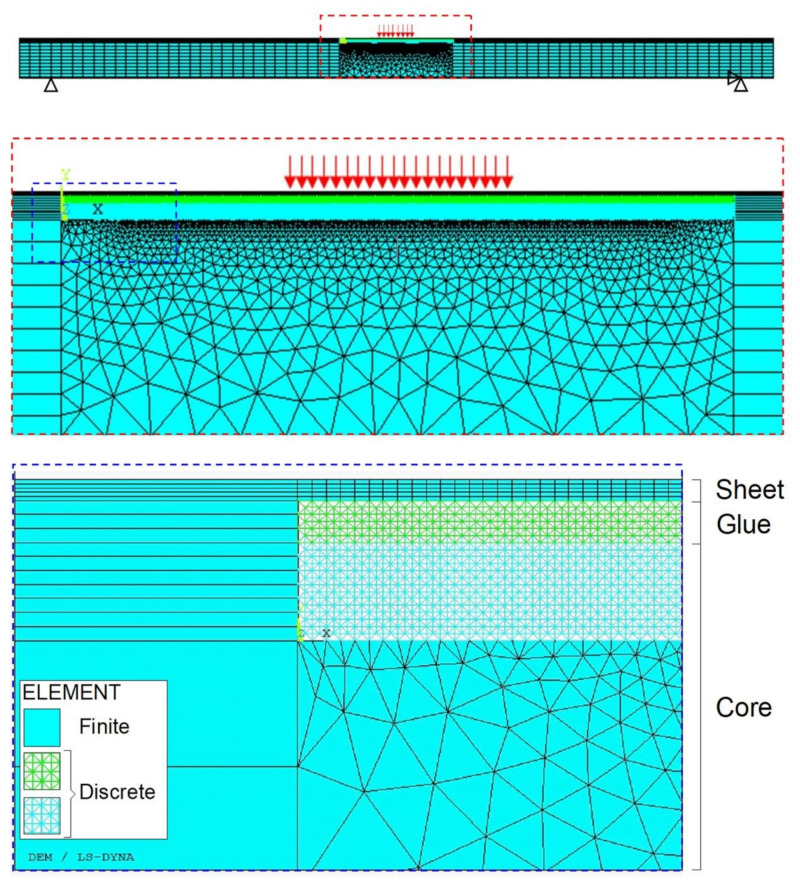
LDEM-DYNA model: discretisation of the sandwich panel under three-point bending.

A 2D model is employed, as the examined configuration is characterised by a plain strain condition. The discretisation adopted is shown in Figure 7, together with the reference system Oxyz.

Two different discretisations are employed in the same hybrid model:

(a) In the central region of the specimen (covering a length equal to 300 mm):
  - the sheets (up and down layers) are modelled by means of FEs (SOLID164);
  - only the upper layer of the glue is modelled, by means of DEs;
  - the core is modelled by means of both DEs and FEs (SOLID164).
  Perfect adhesion is assumed between the above layers;
(b) Outside the central region of the specimen, only the sheets and the core are modelled:
  - the sheets (up and down layers) are modelled by means of FEs (SOLID164);
  - the core is modelled by means of FEs (SOLID164).
  Perfect adhesion is assumed between the above layers.

The lattice discrete element model consists of a symmetric distribution of 45,621 spring elements. In more detail, 300 cubic modules are employed in the horizontal direction (*x*-axis), 10 modules in the vertical direction (*y*-axis), and one module in the thickness direction (*z*-axis). The cubic module length $L_{n}$ is assumed to be equal to 1 mm.

The finite element model consists of 12,811 elements.

Note that the LDEM is limited to some regions since it is where cracks are expected to occur, in accordance to the experimental observations. Moreover, the glue is modelled in correspondence to the LDEM region where both damage and rupture are expected.

The vertical displacement is fixed in correspondence to the two supports in the lower part of the specimen. The horizontal displacement is also fixed in correspondence to one support. Moreover, a prescribed vertical displacement is applied in the upper part of the middle span region, in accordance with the experiments.

Note that three different analyses have been performed, named NT1, NT2, and NT3, respectively, in order to analyse the influence of different $G_{f}$ random fields on the numerical model.

#### 5.1.2. Calibration of the Numerical Model

As far as the parts simulated by FEM are concerned, a linear elastic behaviour is assumed for both the skins and the core (Table 1).

On the other hand, as far as the parts simulated by LDEM are concerned, the constitutive law for each bar element is deduced as is described in Section 3 (Table 1). More details are reported in the following.

Regarding the *polystyrene foam core*, the fracture toughness is computed by means of the relationship between the specific fracture energy value $G_{f}$ (equal to 133 N/m [40]) and the elastic modulus, thus obtaining $K_{c}=0.0651\;\mathrm{MPa}\sqrt{m}$. Then, a stress brittleness number s=0.93 is obtained from Equation (15) (note that the core height is assumed as the structure characteristic dimension), and an equivalent length $d_{eq}=0.00864\;m$ is obtained from Equation (16). Finally, the critical strain, $\varepsilon_{p}=2.42\;\times 10^{-2}$, and the ultimate strain, $\varepsilon_{u}=1.32\;\times 10^{-1}$ for longitudinal elements and $\varepsilon_{u}=1.52\;\times 10^{-1}$ for diagonal elements, are computed by means of Equations (12) and (13), respectively.

Regarding the *glue*, the fracture toughness is computed by means of the relationship between the specific fracture energy $G_{f}$ (equal to 3324 N/m [63]) and the elastic modulus, thus obtaining $K_{c}=0.78\;\mathrm{MPa}\sqrt{m}$. Then, a stress brittleness number s=0.98 is obtained from Equation (15) (note that the glue layer height is assumed as the structure characteristic dimension, and the ultimate tensile strength is employed instead of the yield stress), and an equivalent length $d_{eq}=0.00363\;m$ is obtained from Equation (16). Finally, the critical strain, $\varepsilon_{p}=7.50\;\times 10^{-2}$, and the ultimate strain, $\varepsilon_{u}=1.77\;\times 10^{-1}$ for longitudinal elements and $\varepsilon_{u}=2.04\times \;10^{-1}$ for diagonal elements, are computed by means of Equations (12) and (13), respectively.

#### 5.1.3. Results and Discussion

The numerical load–displacement curves obtained for the simulations NT1, NT2, and NT3 are plotted in Figure 5b.

The maximum values of load and vertical displacement are equal to 11.68 kN, 10.53 kN, 12.17 kN, and 74.14 mm, 66.67 mm, 77.34 for numerical test No. NT1, NT2, and NT3, respectively. Consequently, the average values of peak load and maximum deflection result 11.46 kN (with a standard deviation equal to 0.69 kN) and 72.72 mm (with a standard deviation equal to 4.47 mm). Note that the numerical results are in good agreement with the experimental observations in terms of both peak load and maximum deflection, with errors equal to 5.3% and 4.5%, respectively.

**Figure 8:**
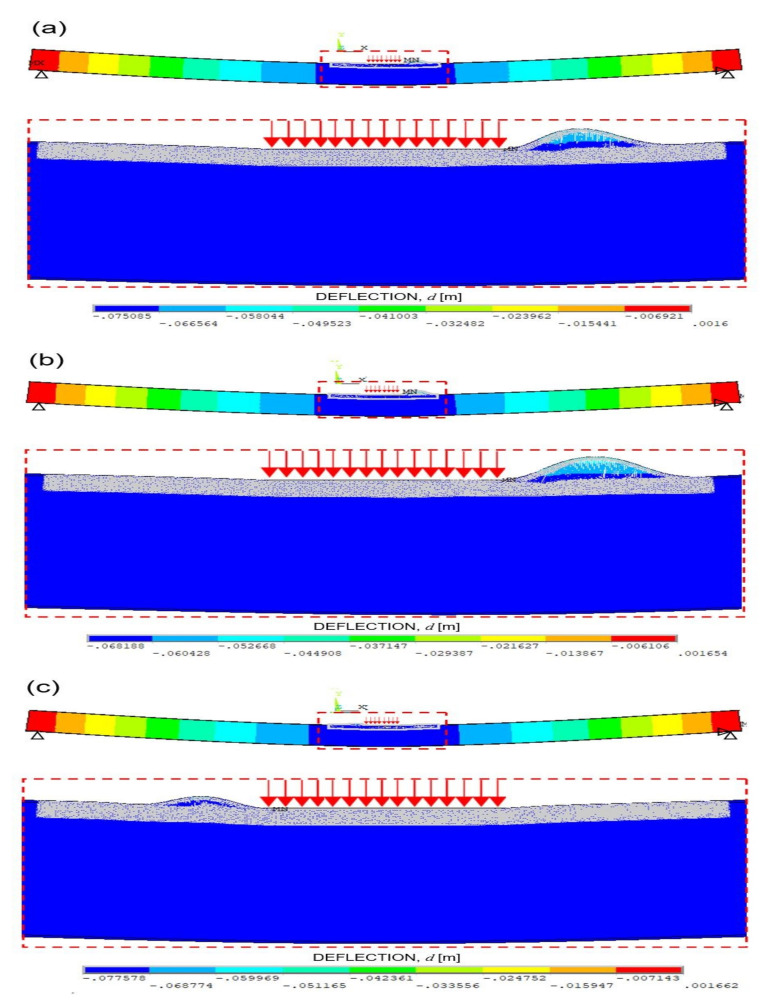
Failure modes obtained by the numerical simulations No.: (**a**) NT1, (**b**) NT2, and (**c**) NT3.

**Figure 9:**
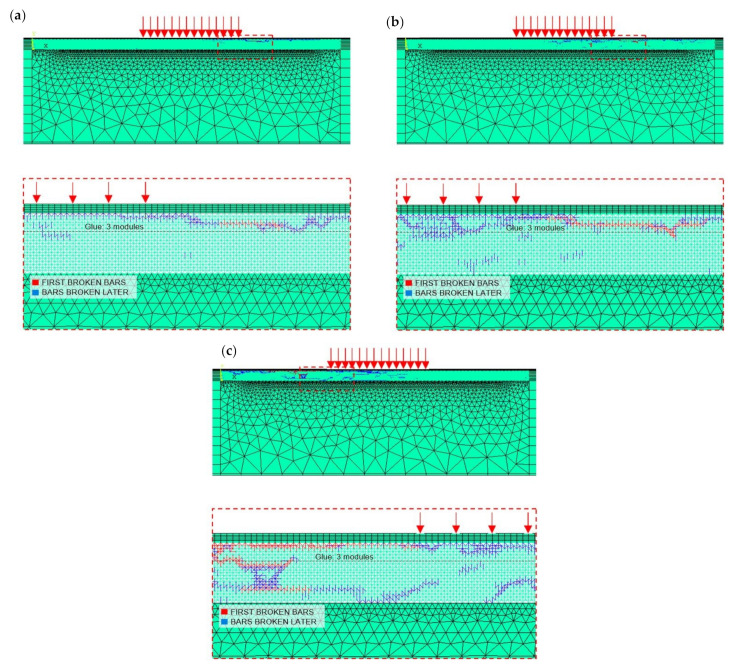
Broken bars obtained by the numerical simulations No.:(**a**) NT1, (**b**) NT2, and (**c**) NT3.

Furthermore, the failure modes observed in the numerical simulations are shown in Figure 8a–c for the test No. NT1, NT2, and NT3, respectively, whereas the broken bars are shown in Figure 9a–c for the test No. NT1, NT2, and NT3, respectively.

In Figure 8**,** it can be observed that the different $G_{f}$ random field, that characterises each numerical simulation, does not affect the general behaviour of the model, being the failure mechanisms always represented by the skin buckling.

First, the bar elements highlighted in red break. Such bars are located within the glue layer (simulation NT1) or within both the glue and the core (simulations NT2 and NT3) at the edge of the zone where the prescribed vertical displacement is applied. The buckling of the upper skin is the failure mechanism related to this first break. Note that such a failure mechanism is experimentally observed in test No. T1 (see Figure 5a).

Subsequently, the final rupture is reached when the bar elements highlighted in blue break. In simulations NT1 and NT2, such bars are mainly located within the glue layer. In these cases, the indentation of the upper skin may be recognised. Note that such a failure mechanism is experimentally observed in tests No. T1 and T2 (see Figure 6a,c). Moreover, the damage related to the blue bars is more homogeneously spread within both the core and the glue in simulation NT3. In general, it can be stated that the final rupture is caused by the core failure in the zone surrounding the prescribed vertical displacement, in accordance with the experimental observations.

Note that the different $G_{f}$ random fields associated to the numerical simulations do not affect the general behaviour of the model. However, the sensitivity of the model to the randomness allows to reproduce the natural scatter of experimental testing, in terms of both peak load and maximum deflection values, damage location, and failure mechanisms.

### 5.2. Four-Point Bending Testing

In this section, the flexural behaviour of sandwich panels subjected to four-point bending tests is simulated by following the same procedure discussed in Section 5.1.

The sandwich panel has the geometry shown in Figure 4a.

#### 5.2.1. Numerical Model Description

Two different 2D models are employed. Due to the symmetry condition along the mid span vertical axis, a half of the specimen is modelled by using two different discretisations, as is detailed in the following.

**Figure 10:**
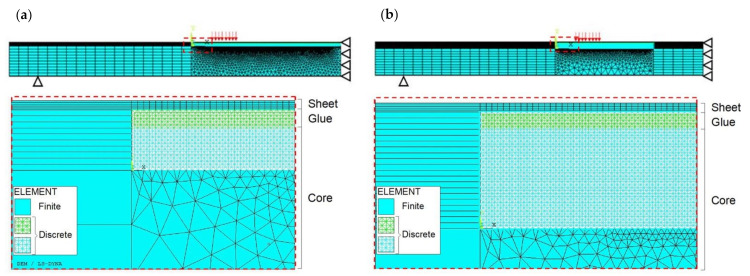
LDEM-DYNA model: discretisation of sandwich panel under four-point bending testing: (**a**) model A and (**b**) model B.

Model A, shown in Figure 10a, is characterised by the following:

(i) A region on the right-hand side, covering a length equal to 450 mm, where:
  - the sheets (up and down layers) are modelled by means of FEs (SOLID164);
  - only the upper layer of the glue is modelled, by means of DEs;
  - the core is modelled by means of both DEs and FEs (SOLID164).
  Perfect adhesion is assumed between the above layers.
(ii) A region on the left-hand side (outside the above one), where only the sheets and the core are modelled:
  - the sheets (up and down layers) are modelled by means of FEs (SOLID164);
  - the core is modelled by means of FEs (SOLID164).
  Perfect adhesion is assumed between the above layers.

The lattice discrete element model consists of 68,421 spring elements. In more detail, 450 cubic modules are employed in the horizontal direction (*x*-axis), 10 modules in the vertical direction (*y*-axis), and one module in the thickness direction (*z*-axis). The cubic module length $L_{n}$ is assumed to be equal to 1 mm.

The finite element model consists of 26,992 elements.

Such a hybrid model is that verified in Section 5.1.3 for the case of three-point bending.

Model B shown in Figure 10b is characterised by two different discretisations:

(i) In the central region (covering a length equal to 300 mm):
  - the sheets (up and down layers) are modelled by means of FEs (SOLID164);
  - only the upper layer of the glue is modelled by means of DEs;
  - the core is modelled by means of both DEs and FEs (SOLID164).
  Perfect adhesion is assumed between the above layers.
(ii) Outside the central region, only the sheets and the core are modelled:
  - the sheets (up and down layers) are modelled by means of FEs (SOLID164);
  - the core is modelled by means of FEs (SOLID164).
  Perfect adhesion is assumed between the above layers.

The lattice discrete element model consists of 90,641 spring elements. In more detail, 300 cubic modules are employed in the horizontal direction (*x*-axis), 20 modules in the vertical direction (*y*-axis), and 1 module in the thickness direction (*z*-axis). The cubic module length $L_{n}$ is assumed to be equal to 1 mm.

The finite element model consists of 11,685 elements.

Both A and B models are constrained as follows. The vertical displacement is fixed in correspondence to the supports in the lower part of the panel. The horizontal displacement is fixed in correspondence to the middle-cross section for the symmetry condition. Moreover, a prescribed vertical displacement is applied in the upper part.

Note that three different analyses are performed for each model (named NF1, NF2, and NF3 for A model, and NF4, NF5, and NF6 for B model), in order to examine the influence of different $G_{f}$ random fields on the numerical model. Note that the $G_{f}$ random fields employed in simulations NF1, NF2, and NF3 are also adopted for simulations NF4, NF5, and NF6, respectively.

#### 5.2.2. Calibration of the Numerical Model

The input data are reported in Table 1. The parameters of both the LDEs and FEs are those shown in Section 5.1.2.

#### 5.2.3. Results and Discussion

**Figure 11:**
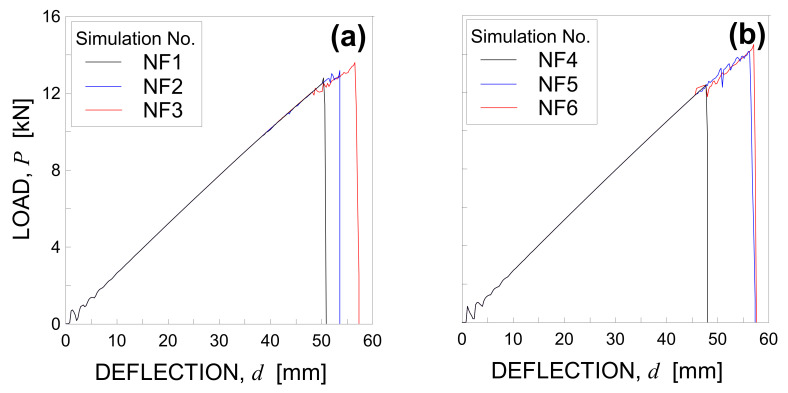
Load-deflection curve under four-point bending obtained by using (**a**) model A and (**b**) model B.

The numerical load-displacement curves obtained for simulations NF1, NF2, and NF3, and NF4, NF5, and NF6 are plotted in Figure 11a,b, respectively.

For A simulations, the average values of peak load and maximum deflection result 13.19 kN (with a standard deviation equal to 0.33 kN) and 53.51 mm (with a standard deviation equal to 2.51 mm), respectively.

For B simulations, the average values of peak load and maximum deflection result 13.67 kN (with a standard deviation equal to 0.91 kN) and 53.60 mm (with a standard deviation equal to 4.17 mm), respectively. It can be noticed that the results of model B are in good agreement with those of model A in terms of both peak load and maximum deflection, with errors equal to 3.69% and 0.17%, respectively.

**Figure 12:**
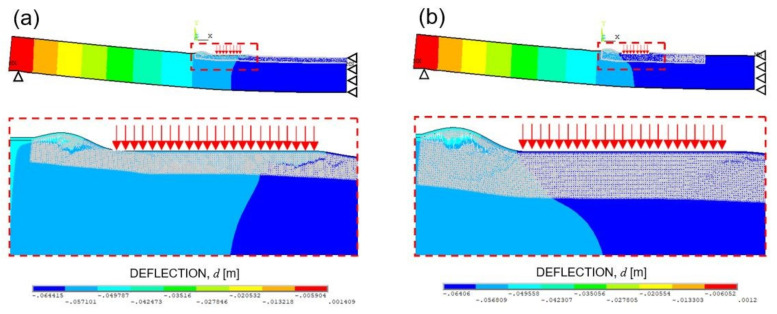
Failure modes observed in the numerical simulation No.: (**a**) NF1 and (**b**) NF4.

**Figure 13:**
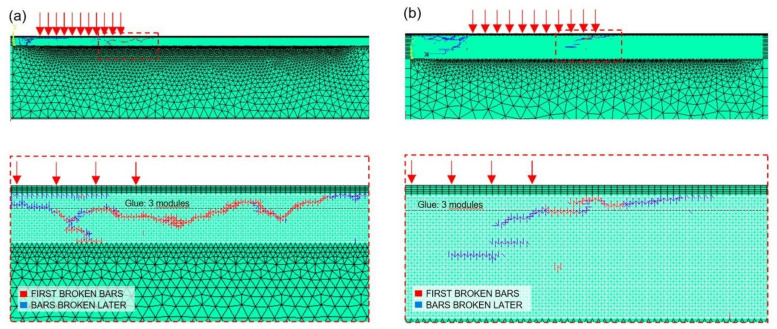
Broken bars in the numerical simulation No.: (**a**) NF1 and (**b**) NF4.

Furthermore, the failure modes observed in the numerical simulations NF1 and NF4 (characterised by the same $G_{f}$) are shown as examples in Figure 12a,b, whereas the broken bar elements for the same simulations are shown in Figure 13a,b.

In Figure 12, it can be observed that the failure mechanisms are always represented by the skin buckling, even when the GF random field, that characterises each numerical simulation, is changed. Such a trend is the same highlighted for the three-point bending test simulations (see failure mechanisms reported in Figure 8).

By considering the results of all models, it can be stated that the bars that break first are within the glue layer (simulations NF2, NF3, NF4, NF5, and NF6) or within both the glue and the core (simulation NF1) surrounding the edge of the zone where the prescribed vertical displacement is applied. The buckling of the upper skin is the failure mechanism related to this first break.

Subsequently, the final rupture is reached when the bar elements highlighted in blue break. In all the simulations performed, such bars are distributed in the area beneath the applied vertical displacement, thus highlighting the presence of the indentation of the upper skin. Then, the final rupture is caused by the core failure in the zone surrounding the zone where the prescribed vertical displacement is applied. Such a failure mechanism is also pointed out in Section 5.1.3 for the simulations related to three-point bending testing.

It can be noticed that the LDEM-DYNA model of sandwich panels subjected to four-point bending testing is able to correctly reproduce the failure mechanisms observed in the experimental tests, which are typical failure modes for sandwich panels with foam core. Moreover, failure modes observed in B simulations are similar to those in A simulations. Therefore, it seems that model B is able to reproduce the four-point bending problem with the same accuracy but with lower computational costs with respect to A model.

Moreover, as has previously been observed in the simulations of three-point bending problem (see Section 5.1.3), different $G_{f}$ random fields associated to the numerical simulations allow us to reproduce the natural scatter of experimental testing, in terms of both peak load and maximum deflection values, damage location, and failure mechanisms.

## 6. Conclusions

In the present research work, the fracture behaviour of sandwich panels has been numerically and experimentally analysed. The numerical model employed is a novel hybrid model, that is, a model developed implementing the LDEM in the Ansys LS-DYNA finite element code. It allows us to simulate the degradation of the material by considering the breaking of the discrete elements in the LDEM region.

Three-point bending tests, carried out at the University of Parma on sandwich panels, have been simulated by means of the above hybrid model. Such a model is able to reproduce the actual failure mechanisms of the tested panels. More precisely, a final rupture caused by the core failure has been deduced through these simulations, in accordance to the experimental observations.

Due to the promising numerical results obtained, the same procedure has been employed to simulate four-point bending tests. Such simulations have provided the same failure mechanisms observed in the experimental three-point bending tests, which are typical failure modes for sandwich panels with foam core.

Moreover, the randomness included in the model allows us to reproduce the natural scatter of experimental testing, in terms of both peak load and maximum deflection values, damage location, and failure mechanisms, without altering the general behaviour of the model.

## Figures and Tables

**Table 1:** Material properties of sandwich panel constituents.

Sandwich Const.	Ref.	E	ν	ρ	$\sigma_{p}\;[\mathrm{MPa}]$	$G_{f}\;[N/m]$
Core	[37,61]	28	0.30	40	0.7	133
Sheets	[61]	7000	0.33	1300	-	-
Glue	[61,63]	162	0.25	1500	13	3324

## Nomenclature

$A_{d}$	cross-sectional area of diagonal bar elements
$A_{n}$	cross-sectional area of longitudinal bar elements
$A_{i}^{*}$	equivalent fracture area of the *i*-th bar element
$C_{ij}$	damping matrix
$C_{p}$	velocity wave
$d_{eq}$	equivalent length of the material
E	Young’s modulus
$F_{i}\left(t\right)$	internal nodal forces
$G_{f}$	fracture energy
$G_{f}$	fracture energy field
$K_{c}$	fracture toughness
$L_{d}$	lengths of diagonal bar elements
$L_{i}$	length of the *i*-th bar element
$L_{n}$	lengths of longitudinal bar elements
m	mass of the basic cubic modulus
$M_{ij}$	mass matrix
$P_{i}\left(t\right)$	external nodal forces
q	critical crack length
$R_{e}$	structure characteristic size
s	stress brittleness number
${\dot{x}}_{j}$	nodal velocity
${\ddot{x}}_{j}$	nodal acceleration
$\Delta t_{max}$	maximum time-interval for integration
$\varepsilon_{p}$	critical strain
$\varepsilon_{u}$	ultimate strain
ν	Poisson ratio
ρ	mass density
$\sigma_{p}$	material strength (tensile strength)
